# Generalized prurigo nodularis after dermatomal herpes zoster: Resolution with nemolizumab

**DOI:** 10.1016/j.jdcr.2025.04.001

**Published:** 2025-04-14

**Authors:** Claire Fason, Stephen Tyring

**Affiliations:** a McGovern Medical School, University of Texas Health Science Center, Houston, Texas; b Department of Dermatology, McGovern Medical School, University of Texas Health Science Center, Houston, Texas; c Center for Clinical Studies, Houston, Texas

**Keywords:** herpes zoster, nemolizumab, prurigo nodularis, pruritus

## Introduction

Prurigo nodularis (PN) is a chronic skin disorder that classically presents as intensely pruritic nodules. PN is a clinical diagnosis, but upon histopathological studies, nodules show orthohyperkeratosis, irregular epidermal hyperplasia along with dense dermal infiltrates consisting of increased T lymphocytes, mast cells, and eosinophilic granulocytes.^1^ Although the etiology is unknown, it is thought to be a cutaneous reaction from chronic itch and scratching. PN is associated with various comorbid conditions that often involve chronic pruritus including atopic dermatitis, HIV, chronic kidney disease, and various hematologic malignancies.^2^ Herpes zoster, commonly known as shingles, is caused by the reactivation of latent varicella zoster virus. Herpes zoster presents with burning pain and a vesicular rash usually in one dermatome.^3^ Post herpetic neuralgia is a common sequela of herpes zoster, but pruritus may also occur after zoster. One report suggests that both PN and herpes zoster are predictive of HIV infection.^4^ However, a literature search on www.pubmed.gov revealed no reported cases of widespread PN after herpes zoster. PN in the same dermatome has been documented after herpes zoster.^5^ We present 2 cases of widespread PN after dermatomal herpes zoster infection successfully treated with nemolizumab, an interleukin (IL) 31 receptor antagonist that was recently US Food and Drug Administration approved to treat PN.^6^

## Case report

### Case 1

**Fig 1:**
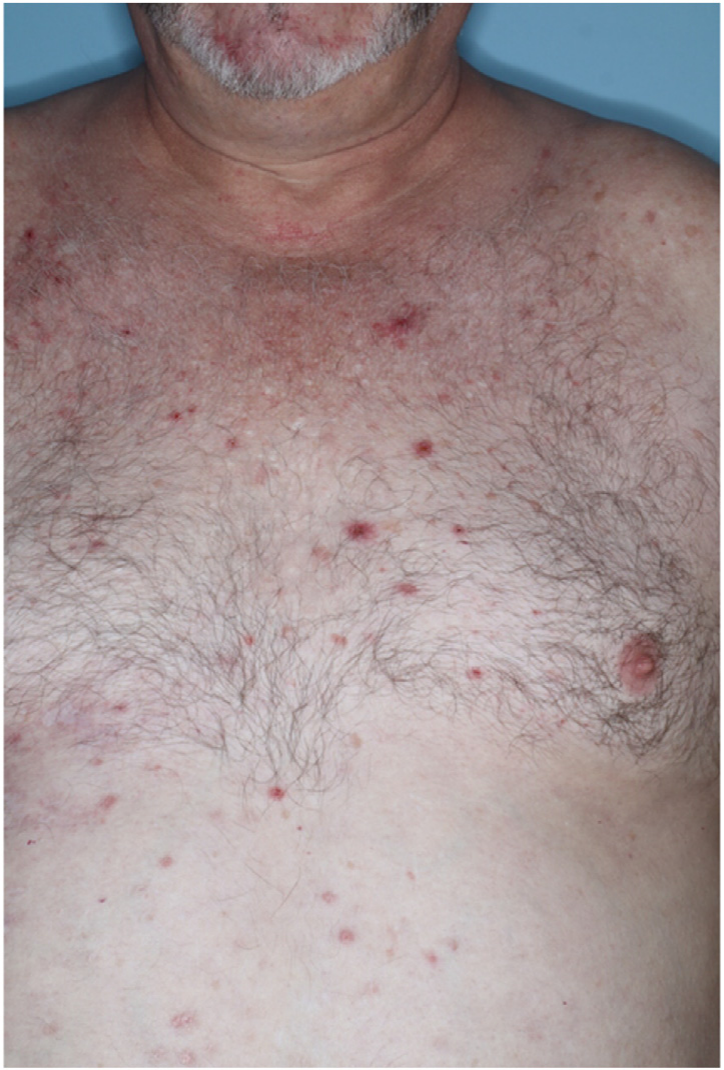
Chest of patient 1: prurigo nodularis before starting nemolizumab therapy.

**Fig 2:**
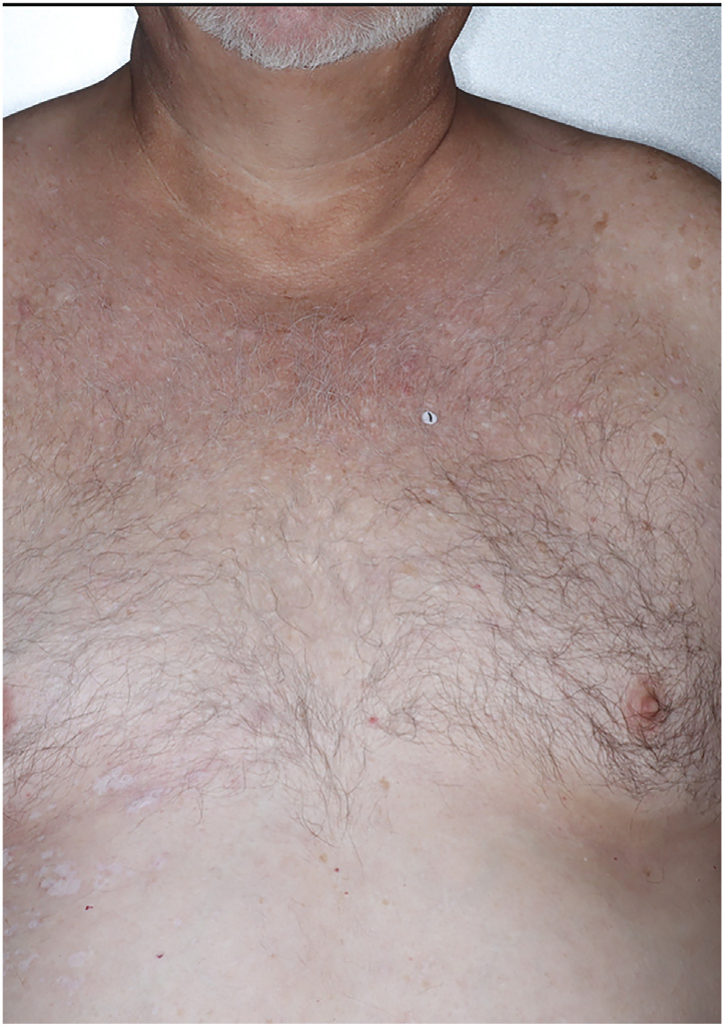
Chest of patient 1: clearance after 2 months of nemolizumab therapy.

A 54-year-old man presented to clinic with a history 2 months previously of a blistering, painful, and pruritic rash on his abdomen located on the right T6 anterior and posterior dermatome, which was consistent with herpes zoster. He had been treated with valacyclovir, prednisone, topical triamcinolone 0.1% ointment, and lidocaine 5% topical ointment, but the pruritus continued along with a few pruritic papules. Upon follow-up 1 month later, papules and nodules were present on the T6 dermatome, and new papules were noted on his left and right inferior eyelid. He was given 1% trifluridine eye drops. Two months after the patient’s initial presentation he complained of intensely pruritic nodules located throughout his bilateral upper and lower extremities, chest, abdomen, and back (Fig 1). Biopsy of these nodules showed hyperkeratosis, acanthosis, and hypergranulosis present in the epidermis with underlying thickening of that papillary dermis, consistent with the diagnosis of PN. Although he denied risk factors for HIV, he was tested and found to be seronegative. The patient received 60 mg of nemolizumab via subcutaneous injection every 4 weeks. The patient’s pruritus resolved within 1 month after the first dose, and the nodules resolved 1 month after the second dose (Fig 2). After this dose, the patient started nemolizumab 30 mg monthly, which he continued to receive for 3 months before discontinuation. Thus far, his PN has not recurred.

### Case 2

**Fig 3:**
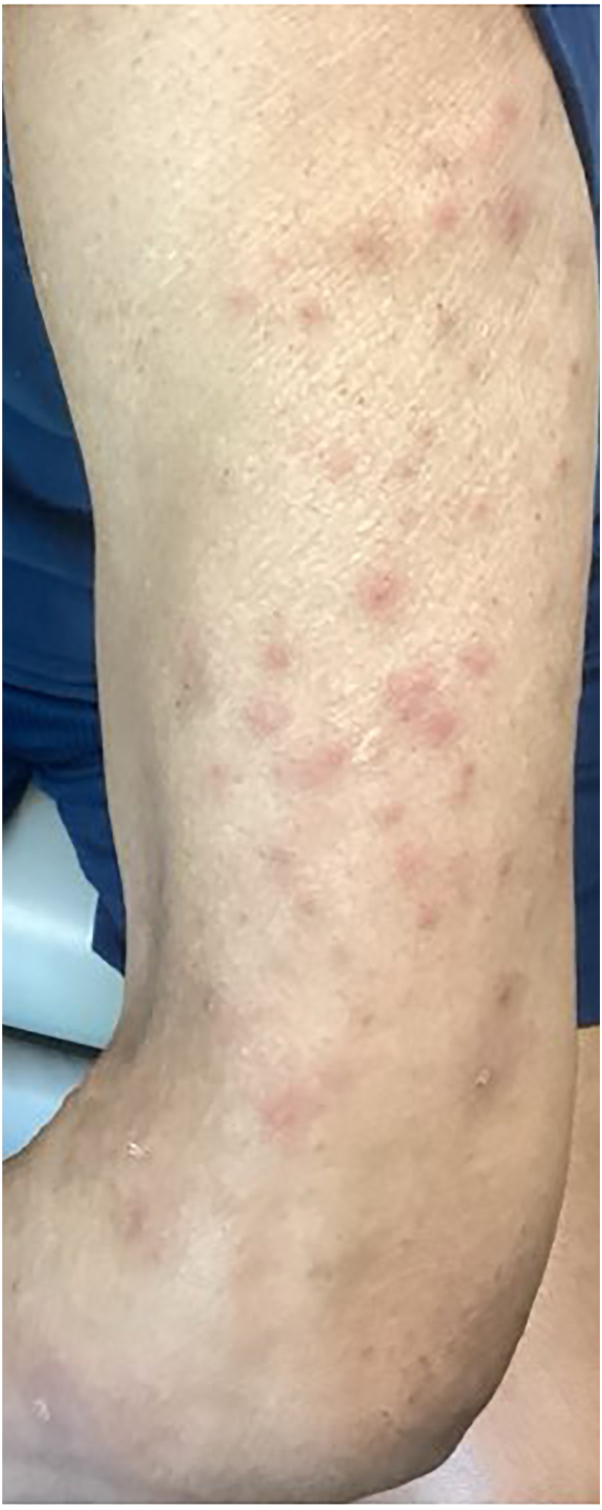
Left arm of patient 2: prurigo nodularis before nemolizumab therapy.

**Fig 4:**
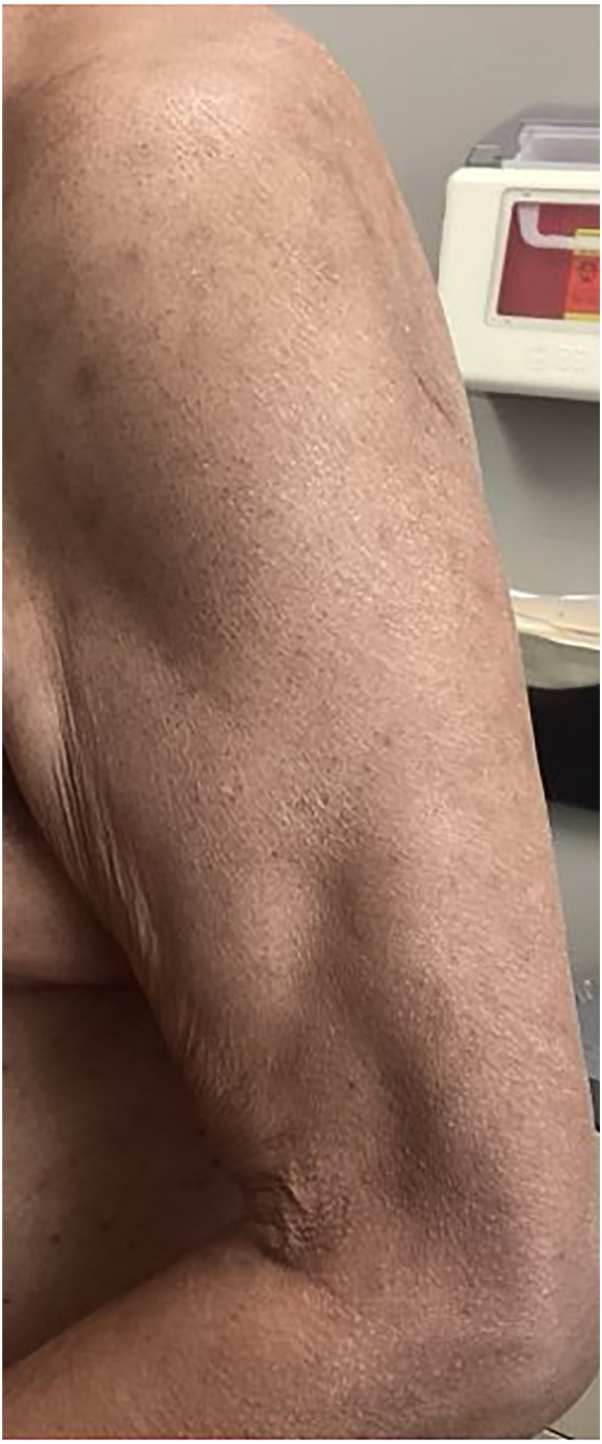
Left arm of patient 2: complete response after 2 months of nemolizumab therapy.

A 69-year-old man with a significant past medical history of heart transplantation presented to clinic with erythematous vesicles consistent with herpes zoster located on his left anterior and posterior C6 and C7 dermatomes. He was prescribed gabapentin 600 mg daily, valacyclovir 1 g 3 times daily for 7 days, and topical 5% lidocaine 3 times daily. One month later the patient complained of severe pruritus and pain triggered by light touch to the dermatome. Doxepin 25 mg 1 to 2 nightly and clobetasol 0.05% ointment 3 times daily were added to his therapy. Two months after presenting with herpes zoster the patient presented to clinic with numerous pruritic nodules on his chest, abdomen, upper, and lower extremities (Fig 3). The lesions were consistent with the clinical diagnosis of PN, therefore biopsy was deferred. Because of the patient’s history of heart transplant, nemolizumab was discussed with his transplant team. The team noted that the patient had tested negative for HIV and cleared him for nemolizumab therapy. He received a subcutaneous injection of 60 mg of nemolizumab. One month later he received his second dose of nemolizumab 60 mg, at this visit his pruritus had resolved, gabapentin was tapered off during the subsequent 2 weeks and doxepin was discontinued. No new PN lesions were seen, and previous PN lesions were beginning to resolve. After 2 monthly doses of 60 mg nemolizumab, no prurigo nodules remained, only postinflammatory hyperpigmentation (Fig 4). Therefore, he was continued on 30 mg of nemolizumab monthly. Attempts to discontinue nemolizumab have been unsuccessful, because of PN recurrence.

## Discussion

PN is a chronic skin disorder characterized by intensely pruritic nodules. PN is associated with significantly impaired quality of life due to unrelenting pruritus, which leads to sleep disturbances, interference with daily life, and mood disturbances.^7^ Although the etiology of PN is not completely clear, it is believed to be associated with an itch-scratch cycle and related neuroimmune dysregulation. Upon histologic examination, lesions demonstrate increased numbers of immune cells, which release inflammatory cytokines, such as IL-31, as well as disrupted neural architecture.^1^^,^^8^ Although associations exist between PN and other conditions that cause increased pruritus, there is not a known relationship between herpes zoster and PN. Although there is a published case documenting a 60-year-old woman who experienced localized PN in the same dermatomal distribution as her prior herpes zoster infection, a PubMed search found no other cases of widespread PN after dermatomal herpes zoster.^5^ Although the connection between herpes zoster and PN is not well understood, the pathogenesis of herpes zoster and PN both implicate neuronal and cytokine involvement. IL-31 is strongly implicated in the pathogenesis of PN, and there is a known correlation between IL-31 and HIV related pruritus.^9^ Although there is no reported association between herpes zoster and IL-31, herpes zoster is known to be associated with an increased level of inflammatory cytokines, including IL-4, IL-6, and IL-10.^10^ It is possible that, similar to HIV associated pruritus, IL-31 is involved in post herpetic pruritus. Another possible explanation of the association between PN and herpes zoster is dermatomal itching, which is common in healing herpes zoster. This may lead to the tendency to respond to the pruritus outside of the dermatome, which in turn leads to the development of PN.

Both patients experienced generalized PN outside of the dermatome affected by their herpes zoster infection and both were successfully treated with nemolizumab, an IL-31 receptor antagonist. Case 2 documents the first patient with a history of organ transplantation treated with nemolizumab. The pathophysiology of generalized PN after herpes zoster is not well understood. Therefore, the relationship between these conditions is only known to be temporal.

## Conflicts of interest

None disclosed.
